# Stem Cells in Veterinary Medicine—Current State and Treatment Options

**DOI:** 10.3389/fvets.2020.00278

**Published:** 2020-05-29

**Authors:** Metka Voga, Neza Adamic, Modest Vengust, Gregor Majdic

**Affiliations:** ^1^Faculty of Veterinary Medicine, University of Ljubljana, Ljubljana, Slovenia; ^2^University of Ljubljana, Ljubljana, Slovenia

**Keywords:** stem cells, clinical veterinary medicine, regenerative medicine, dogs, cats, horses

## Abstract

Regenerative medicine is a branch of medicine that develops methods to grow, repair, or replace damaged or diseased cells, organs or tissues. It has gained significant momentum in recent years. Stem cells are undifferentiated cells with the capability to self—renew and differentiate into tissue cells with specialized functions. Stem cell therapies are therefore used to overcome the body's inability to regenerate damaged tissues and metabolic processes after acute or chronic insult. The concept of stem cell therapy was first introduced in 1991 by Caplan, who proposed that massive differentiation of cells into the desired tissue could be achieved by isolation, cultivation, and expansion of stem cells in *in vitro* conditions. Among different stem cell types, mesenchymal stem cells (MSC) currently seem to be the most suitable for therapeutic purposes, based on their simple isolation and culturing techniques, and lack of ethical issues regarding their usage. Because of their remarkable immunomodulatory abilities, MSCs are increasingly gaining recognition in veterinary medicine. Developments are primarily driven by the limitations of current treatment options for various medical problems in different animal species. MSCs represent a possible therapeutic option for many animal diseases, such as orthopedic, orodental and digestive tract diseases, liver, renal, cardiac, respiratory, neuromuscular, dermal, olfactory, and reproductive system diseases. Although we are progressively gaining an understanding of MSC behavior and their mechanisms of action, some of the issues considering their use for therapy are yet to be resolved. The aim of this review is first to summarize the current knowledge and stress out major issues in stem cell based therapies in veterinary medicine and, secondly, to present results of clinical usage of stem cells in veterinary patients.

## Types of Stem Cells

By definition, stem cells are undifferentiated cells capable of self—renewal and transformation into different specialized cells (1). They are classified by their source as (a) embryonic (ESC), (b) adult, and (c) induced pluripotent stem cells (IPSC) (2, 3). Considering their phase of development and differentiation, they are further classified as totipotent, pluripotent, or multipotent cells (4).

Totipotent stem cells are present only in a very early embryo during the morula stage before gastrulation starts. They are capable of developing into all embryonic and extra-embryonic tissues. Subsequent divisions of cells during early embryonic development lead to the emergence of the blastocyst with pluripotent ESC being present in the inner cell mass. ESC can give rise to all tissue cells in the body, with the exception of extra-embryonic tissues and germ cells (2, 5). With further cell development, pluripotent ESC gradually lose their pluripotency and become multipotent. The multipotent stage is characterized by the ability of cells to differentiate into limited types of specific cells, often depending on their germ layer origin (6).

The first isolation of human ESC was reported in 1998 (7). This triggered numerous studies about gene expression and function during embryonic development and cell differentiation processes, as well as attempts to identify gene targets for new drugs that might be useful in tissue regeneration therapies. However, broad-spectrum therapeutic capabilities of human ESC collided with ethical, moral, and cultural dilemmas because their harvesting is associated with the destruction of human embryos. Other sources of stem cells, therefore, had to be explored to continue the research into stem cell—based therapies. One alternative was developed in 2006 by Takahashi and Yamanaka, who reprogrammed adult mouse fibroblasts into pluripotent stem cells by retroviral transduction of four specific genes: OCT4, c-Myc, SOX2, and KLF4. These cells were termed IPSC and are similar to the ESC in their morphology, growth properties, and in the expression of ESC marker genes. Although the discovery of IPSC was remarkable progress in stem cell therapy, retroviral transductions can create chromosomal alterations, which increase the risk of tumorigenesis, raising questions about the safety of IPSC for regenerative medicine (3).

Another alternative to ESCs presents the stem cells which are present in the adult organism. Bone marrow and umbilical cord blood contain hematopoietic stem cells (HSCs) and non-hematopoietic or mesenchymal stem cells (MSC), the latter residing also in numerous other tissues. These cells are multipotent because they can differentiate into specific body cell types. HSCs can differentiate into different cells of the immune system, erythrocytes and platelets, and MSCs into cells of bone, cartilage, ligaments, tendons, fat, skin, muscle, and connective tissue. MSCs are activated endogenously when needed to replace dead, injured, or diseased tissue cells (8). The first mention of adult multipotent cells/MSC dates to 1968 when the osteogenic population of cells with fibroblast-like morphology was isolated from the bone marrow (9). Early studies showed that multipotent stem cells are capable of differentiating into osteoblasts, chondroblasts, and adipocytes (10). This leads to the belief that MSCs show their therapeutic potential through differentiation into tissue cells (11, 12). However, numerous subsequent studies have questioned this and today it is believed that the primary mechanism of MSC regenerative abilities stems from their immunomodulatory and tissue repair mechanisms. It is presumed that perivascular localization of MSC in various tissues plays an essential role in enabling these cells to detect local or distant tissue damage and respond to it by directed migration to the site of injury and participation in the healing process (13). Based on this, Caplan proposed that the term “mesenchymal stem cells” should be changed into “medicinal signaling cell” (MSC) (14).

Compared to other stem cell types, MSCs are recognized as the most promising stem cell type for stem cell therapy due to the simple procedures needed for their harvest, isolation, high cell yield upon their harvesting, and the lack of ethical restraint when in use. To prevent the confusion in the field of adult stem cells research, The Mesenchymal and Tissue Stem Cell Committee of the International Society for Cellular Therapy (ISCT) proposed a set of standards to define human MSC for both laboratory-based scientific investigations and pre-clinical studies (15). In essence: (1) MSC must be plastic-adherent when maintained in standard culture conditions using tissue culture flasks, (2) 95% of the MSC population must express CD105, CD73, and CD90 and lack the expression of CD45, CD34, CD14 or CD11b, CD79a or CD19, and HLA class II, and (3) MSCs must be able to differentiate into osteoblasts, adipocytes, and chondroblasts under standard *in vitro* differentiating conditions.

## MSC Sources

### Tissue Origin of MSC

To date, MSCs were successfully isolated from various tissues, and based on the source they have different properties, which should be considered when choosing the optimal stem cell therapy approach aiming at the tissue healing. In dogs, horses and cats, the most common companion veterinary patients, MSCs have been isolated from bone marrow (16–23), adipose tissue (16, 17, 19–21, 23, 24), synovium (16), synovial fluid (17, 21, 25, 26), synovial membrane (26), infrapatellar fat pad (16), umbilical cord (27–29), umbilical cord blood (19, 30, 31), Wharton's Jelly (19, 31), muscle and periosteum (20, 32), gingiva and periodontal ligament (33), peripheral blood (34–37), endometrium (38), and placenta (31). In mice, MSCs were also isolated from the brain, spleen, liver, kidney, lung, muscle, thymus, and pancreas (39). Currently, the most commonly used sources of MSC for stem cell therapies are bone marrow and adipose tissue because they offer larger number of MSCs than other tissues. Among the two, the adipose tissue is a particularly attractive source of MSCs due to the minimally invasive procedure needed to obtain cells. Although MSCs isolated from bone marrow and adipose tissue have similar surface immunophenotyping and trilineage differentiation (16, 17, 40), there are important differences in terms of proliferation and differentiation capacity, and their secretory profiles. In some studies, canine adipose tissue derived MSC (ADMSC) were shown to have higher proliferative potential (17, 19, 40, 41), whereas bone marrow derived MSC (BMMSC) exhibited a higher secretory production of soluble factors and exosomes (19, 41). Canine ADMSCs were reported to have superior chondrogenic (17) and osteogenic potential (19) in comparison to BMMSCs, whereas in horses, chondrogenic and osteogenic potential seem to be higher in BMMSC (42, 43). Equine BMMSCs also seem to have a higher migration capacity (21) than ADMSCs. Another potential source of stem cells with high chondrogenic potential might be synovium derived MSCs, as some studies have shown that they are expanding more rapidly than ADMSC in horses (21) and have a greater chondrogenic potential than ADMSC and BMMSC in dogs (16, 17). When choosing adipose tissue as a source of MSCs, anatomical site of harvesting is also important. Guercio et al. (44) reported that subcutaneous ADMSCs have better proliferation potential than ADMSCs derived from visceral fat depots, and Yaneselli et al. (45) reported that subcutaneous ADMSCs remain multipotential in cell culture for a longer time and have higher osteogenic potential. Bahamondes et al. (46) also reported that visceral adipose tissue yields a higher number of MSCs in comparison to subcutaneous adipose tissue.

Since differences in stem cell properties might lead to differences in the success of stem cell therapy, they will have to be explored more closely in the future. Currently, there is no evidence that would generally suggest the preferential tissue source of MSC. This is at least partially due to variability in donors' species, donors' age, and donors' health conditions in different studies. Moreover, lack of standardization for the isolation, culture, and characterization of animal MSC considerably hinders the comparison of results between studies, and the variety of tissue sources are causing problems to set the criteria to define MSC. To date, there are no minimal established criteria for the identification of MSC in animals like criteria in humans (15). While all animal MSC show plastic adherence and differentiation potential, not all express the same panel of surface antigens that has been described for human MSC. Most non-human MSC express CD29 and CD44. However, the expression of CD73, CD90, and CD105 varies depending on the species and strain (47).

### Autologous and Allogeneic MSC

Based on the donor–recipient relationship, stem cells can be classified as autologous, allogeneic, or xenogeneic stem cells. Autologous stem cells are collected from and administered to the same individual, allogeneic stem cells are collected from a donor and used in a recipient of the same species, whereas xenogeneic stem cells are those that are transplanted across species (48). When aiming to choose the most appropriate type of cells for particular stem cell therapy, choosing between autologous vs. allogeneic sources may prove challenging, and advantages and disadvantages for one over the other option should be considered. The isolation and expansion of autologous stem cells are time-consuming and associated with the costly procedure. Moreover, the potency of autologous MSC could be affected by patient age (44, 49–53) and existing disease (54). The need for allogeneic off-the-shelf stem cell products derived from young and healthy donors is, therefore, on the rise.

The main concern with allogeneic stem cell therapy is the possibility that MHC I surface molecules on allogeneic MSCs are recognized by recipient CD8+T cells, leading to direct cytotoxicity of foreign cells. In addition, MHC II molecules can be recognized by recipient CD4+ T cells, leading to either cytotoxic or humoral immune response. MHC molecules could also be subjected to indirect recognition by antigen presenting cells, leading to alloantibody production in B cells (55). Despite promising results regarding the safety of allogeneic MSC, several studies conducted *in vitro* (56, 57) and *in vivo* not only in rodents (58, 59) but also in horses (60–62) and dogs (63), showed immunogenic responses provoked by allogeneic MSC. This has raised some concerns about their presumed immunoprivileged characteristics. Joswig et al. (60), Bertoni et al. (64), and Cabon et al. (63) reported local side effects when the application of allogeneic cells was repeated and proposed that adverse reactions are most likely due to recipient's immune recognition of cells after re-exposure. However, when the effects of single and repeated applications of allogeneic cells for osteoarthritis treatment were compared in horses, no clinically relevant differences were observed in the outcome (65).

In line with contradictory clinical outcomes concerning to the immunogenicity of MSC, conflicting results have also been reported in terms of MHC expression depending on their state, tissue origin, breeds, individual donor, and culture conditions. For example, Menard et al. (66) showed that ADMSCs possess an increased capacity to modulate immune cells and that their phenotypic and transcriptomic profile is consistent with lower immunogenicity in comparison to BMMSC.

Regardless of many positive results of the studies encouraging the use of allogeneic MSC, several studies have confirmed that immunosuppressive properties of MSC do not exclude their immunogenicity. Further research is therefore needed to determine potential mechanisms to regulate MHC expression on MSC and to reach an agreement on the issue of MSC immunogenicity. Autologous stem cells, therefore, remain the most commonly used stem cell source in contemporary veterinary medicine.

## Therapeutic Potentials of MSC

Although stem cells were initially thought to be the source of cells that would differentiate and replace damaged or diseased tissues, it has become evident that the therapeutic properties of MSC are achieved mainly through their immunomodulatory functions, which operate in the interaction with the immune system cells. Complex immunomodulation activity of MSC includes their paracrine action, secretion of extracellular vesicles (ECV), apoptosis mediated immunomodulation, and mitochondrial transfer of membrane vesicles and organelles.

### Paracrine Effects

Increasing evidence suggests that the primary mechanism of action of MSC relies on paracrine signaling which results in functional changes in the immune cells, such as monocytes/macrophages (67), dendritic cells (68), T-cells (69), B-cells (70), and natural killer cells (71). Several factors have been reported to contribute to the immunomodulatory effects of MSC. Among them are well-established effectors such as transforming growth factor-beta (TGF-β), indolamine-2,3-dioxygenase (IDO), prostaglandin E2 (PGE2), interleukin 10 (IL-10), and tumor necrosis factor- (TNF) stimulated gene-6 (TSG-6).

TGF-β is involved in many biological processes such as proliferation and differentiation of cells, embryonic development, wound healing, and angiogenesis (72). It affects migration and homing of MSC (73, 74) and their proliferation and differentiation. TGF-β was shown to induce a switch from inflammatory (M1) to antiinflammatory/regulatory (M2) state of macrophages and thus importantly participates in the induction of the regulatory T-cells (Tregs) (75–79). IDO, a metabolic enzyme, is another soluble factor that is secreted by MSC in an inflammatory environment (70). IDO catalyzes reactions leading to T- and B-cell cycle arrest (80), inhibition of T-cell proliferation and induction of Tregs generation (81), inhibition of B-cells (80), and NK cells (82), and is correlated with bystander differentiation of M2 macrophages (83). PGE2, the major prostaglandin, modulates chemokine production, inhibits the attraction of proinflammatory cells, and enhances differentiation of regulatory cells (84). It is a crucial mediator in NK-cell inhibition (82) and has a role in macrophage polarization toward the M2 phenotype (85). Also, its role was recently demonstrated in the clearance of apoptotic cells by MSC (86). IL-10 is an antiinflammatory cytokine that limits Th1 and Th2 response and accessory functions of macrophage and dendritic cells while inhibiting T-cell expansion (87) and driving the production of Tregs (88). MSCs' secretion of IL-10 is stimulated by an inflammatory environment and contact with T-cells (68, 89). TSG-6 is an inflammation-associated protein with anti-inflammatory and protective properties (90). MSC constitutively express TSG-6, which affects their morphology, the size of ECV, proliferation rate, differentiation potential, survival, and colony-forming unit capacity and is, therefore, crucial in maintaining MSC stemness (91). It was shown that TSG-6 induces the switch from M1 to M2 phenotype and increases the number of Tregs, resulting in relieving the symptoms of inflammatory conditions in experimental models of many diseases (92–95).

MSCs are, therefore, capable of altering the course and consequences of a particular disease through the paracrine effects on an individual's immune response.

### Secretion of Extracellular Vesicles (ECV)

The paracrine action of MSCs is not limited solely to the secretion of soluble factors since MSCs have the capability to transfer various molecules through the extracellular vesicles (ECV). ECVs are vesicles arising from the plasma membrane by outward or inward budding (96). They are carriers of miRNA, mRNA, proteins, and mitochondria that are protected by the membrane. This enables ECVs to move long distances inside the body (97, 98). ECV include exosomes, which are 30–150 nm large plasma membrane coated vesicles of endocytotic origin, microvesicles, which are 100–1000 nm large vesicles of non-endocytotic origin, and apoptotic bodies, 50 nm−5 μm large vesicles released during membrane blebbing of apoptotic cells (99).

MSCs were shown to secrete exosomes and at least three other similarly sized types of ECV (100).

Regarding ECVs mechanisms of action, they seem to be similar to those exhibited by MSC themselves. The study by Hyvarinen et al. (101) demonstrated that ECVs enhance M2 macrophages in the same way as MSC, via PGE2 activation. Further, results from a recent study suggest that ECVs suppress T-cells through TGF-β and adenosine signaling (102). The primary role in increasing Tregs was attributed to TSG-6 from canine ADMSC-derived ECVs used for therapy of induced colitis in mice (92). ECVs were also shown to upregulate the IL-10 production when used to treat a mouse model of sepsis (103). In the pig, mouse, and rat animal models, MSC derived ECVs were reported to be beneficial in the respiratory (104), renal (105), and liver diseases (106), and also in the treatment of osteoarthritis (107), spinal cord injury (108), cerebral ischemia (109), and myocardial infarction (110). ECVs were also used in dogs and horses. Kornicka-Garbowska et al. (111) reported improved angiogenesis and elasticity of damaged tendon in a stallion after treatment with ADMSC-derived microvesicles. In dogs, ECVs were reported to promote vascularization, collagen synthesis, and cutaneous wound healing with better effects than their originator cells (112).

ECVs represent the potential to exploit MSC effects in a cell-free manner, with the main advantage being the avoidance of possible MSC side effects such as immune response and pulmonary embolism upon intravenous (IV) application of MSC (97, 98). Yet, cell-to-cell contact is believed to be important for some MSC immunomodulatory properties (68, 70, 113). When heat-inactivated MSCs without secretome but with the intact membrane integrity were infused IV, they did modulate monocyte function in the same way as control cells, increasing IL-10 levels and reducing IFN-γ levels. The results of this study suggest that immune response after MSC administration is not dependent on their active immunomodulatory activity (114), and contact with MSCs alone is sufficient for some immunomodulatory effects.

However, the lack of standardized techniques for isolation and purification of the ECVs remains the major limitation in ECV research. The most commonly used methods for exosome isolation are ultracentrifugation, ultrafiltration, tangential flow filtration, precipitation and size exclusion chromatography and immunoaffinity based methods. For example, key markers of exosomes are associated with endocytosis (115) and include caveolins, clathrins, transferrin receptors, tetraspanins (CD81, CD63, CD9), Alix and TSG101 (116). Ligands and cargo differ between ECV types leading to the presumption that each type of ECVs has a different function. Lack of standardized methods for exosome isolation leads to the incapacity to separate exosomes from other similarly sized ECV. Moreover, there is presently no standard measurement for ECV purity. Inconsistencies in describing ECVs are, therefore, present in the literature (116, 117). Guidelines of the International Society for Extracellular Vesicles appeal to the researchers to use the generic term “extracellular vesicle” rather than a designation of a specific subtype, which should be carefully defined if used. Furthermore, guidelines suggest that isolation and preparation procedure should be described in detail to allow the replication. Confirmation of ECV function requires demonstration that the effect of ECVs occurs without cell-cell contact, and is not achieved with the soluble, non-ECV associated secreted factors (118).

### Apoptosis-Mediated Immunomodulation

Apoptosis might also play an important role in the immunomodulatory effect of MSC. Phagocytic clearance of dying cells (efferocytosis) takes part not only in resolving inflammation and restoring the function of damaged tissue but also in the adaptive and immune responses in inflamed tissues (119). In a study conducted by Luk et al. (114), heat-inactivated MSC modulated monocyte function in the same way as control cells, resulting in increasing IL-10 levels and reducing IFN-γ levels. The results of this study suggest that immune response after MSC administration is not dependent on their active immunomodulatory activity but is derived from other cells, triggered by MSC presence. Recent evidence also shows that innate immune system cells are determinant in mediating the MSC effect. In particular, it was demonstrated by Galleu et al. that MSCs undergo apoptosis in the presence of cytotoxic cells, namely CD56+ NK cells and CD8+ T-cells, after being IV infused. MSC apoptosis induced by cytotoxic cells is MHC-independent and requires physical contact between MSC and cytotoxic cells. Apoptotic MSCs are then phagocytosed by macrophages that ultimately deliver immunosuppressive activity by producing IDO (120). Similar results were obtained by Cheung et al. (121), where monocytes, engulfed with apoptotic MSC, enhanced the inhibition of T-cell proliferation by producing PGE2. Mechanism of apoptosis derived immunosuppression can be, therefore, predictive in clinical therapies where patients displaying high cytotoxicity would be more responsive to MSC (120, 121). de Witte et al. (122) also showed that MSCs were rapidly phagocytosed in the lungs by monocytes and neutrophils after IV administration in mice. Phagocytosis of MSC induces expression of regulatory phenotype in monocytes and induces their polarization, which in turn modulates an adaptive immune system by inducing T-reg cells.

### Mitochondrial Transfer

The mitochondrial transfer has been proposed as another mechanism of MSC action. In addition to transferring molecules via ECVs, MSCs seem to be capable of intercellular transfer of organelles via tunneling nanotubes. In 2006 the first mitochondria transfer between MSC and somatic cells was observed (123). This study revealed that active transfer of mitochondria from adult stem cells to somatic cells can rescue aerobic respiration in mammalian cells with non-functional mitochondria. In a mouse model of pneumonia, human BMMSC could transfer their mitochondria through the tunneling nanotubes to alveolar macrophages, which led to the enhanced phagocytosis of macrophages and antimicrobial effect of MSC (124). Mitochondria transfer was also demonstrated *in vivo* from systemically administered BMMSC to diabetic nephropathy mice model (125). Since mitochondrial transfer is associated with various physiological and pathological activities, the mitochondrial transfer could be potentially useful for future treatments of many pathological conditions.

## MSC Homing

Besides their complex mechanisms of immunomodulation, one of the key advantages of MSC-based therapies is their ability to home the damaged tissue. MSC homing is tightly correlated with chemical factors such as chemokines, cytokines, and growth factors. One of the main chemical factors involved in MSC migration is a stromal cell derived factor 1 (SDF-1), a chemokine released from damaged tissue, sending chemo-attractive signals for cells expressing CXCR4 receptors on the outer membrane (126). However, CXCR4 in non-activated MSCs is present only at low levels on the cell surface but at higher levels intracellularly. Upon activation, MSCs can quickly translocate CXCR4 molecules to the cell surface, which enables them to follow the migration cues (127). Besides CXCR4, other chemokine receptors have been identified on MSC membranes that are involved in MSC migration, such as CCR6, CCR9, CXCR3, and CXCR6 (128). Another chemical agent upregulated in injured tissues and inflammation is osteopontin (OPN). This cytokine recruits MSC to the sites of injury through ligation to the integrin β1 that is expressed on MSC upon induction by OPN (129). Among growth factors, fibroblast growth factor (130), vascular endothelial growth factor (131), hepatocyte growth factor (132), insulin-like growth factor-1 (133), and TGF-β1 (134) have been shown to affect MSC homing importantly. Mechanical factors such as mechanical strain, shear stress, matrix stiffness, and microgravity are also importantly involved in MSC homing (135). In stem cell therapies, local transplantation of MSC is a desirable method for cell administration. In some instances, however, intraparenchymal injection of MSC may not be possible due to potential invasiveness (136). Systemic applications, of which IV route is the least invasive, are therefore preferred. The homing of MSC after IV application is faced with various obstacles. Firstly, systemically transplanted MSC must first exit the circulation and then migrate to the site of the injury (137). Secondly, the MSCs after IV transplantation are often sequestrated and then cleared from the lungs (122, 138–142). MSC are relatively large cells, with the average size of 30 μm in suspension. In comparison, pulmonary capillaries are, on average, only 14 μm in diameter, which causes the mechanical entrapment of MSC in the lungs (138). In addition to their size, molecular interactions of MSC with the pulmonary endothelium may be another reason for their accumulation in the lungs. Wang et al. (143) were the first to show that the critical cause of MSC entrapment in lung tissue is the excessive expression and activation of integrins. Their study demonstrated that the blockade of integrins resulted in substantially reduced lung entrapment of MSCs in mice, increased levels of circulating MSCs in the blood, and enhanced homing of MSCs toward the target tissues. Monitoring MSCs after systemic infusion also demonstrated that MSCs are short lived and often disappear 24 h after infusion (122, 139). The long-term beneficial effects of MSCs are thus somehow contradictory to their short lifetime (144). Interestingly, their therapeutic effect may not be correlated with cells' viability, as it was shown by de Witte et al. (122) that despite the accumulation of cells in the lungs and short viability after IV administration, MSC exhibit long term effect through the apoptosis and phagocytosis by immune cells.

To avoid problems with IV administration, other routes have been tested, such as intraarterial (IA) and intraperitoneal (IP) administration. IA administration may reduce the accumulation of MSC in the filtering organs and is thus a promising way for stem cell treatment of ischemic injuries (98). IA injection of MSC may allow better distribution of cells. Centralized IA administration of MSC via the femoral artery in an intact porcine model showed increased uptake of MSC in various organs, especially in the liver (98). The downside of IA administration is that it is technically a more challenging procedure than IV injection (145) and there is a risk of a possible intravascular occlusion (145, 146). Sole et al. (145) observed arterial thrombosis in horses when the intraarterial application of allogeneic BMMSC was performed via IA regional limb perfusion. Interestingly, the complication of thrombosis was not detected when performing IA injection without using a tourniquet, indicating that a thrombosis is a consequence of blood stasis and not the MSC application (147). Similarly, IA injection of MSC was proven feasible with allogeneic equine BMMSC injected into the cranial tibial artery in horses, also without a tourniquet (148). Nishimura et al. (149) also proved the safety and efficacy of the IA application of MSCs by administering autologous BMMSCs via the hepatic artery in a canine model of liver fibrosis. IP administration of MSCs is rarely used, but carries the potential to reach intraabdominal sites and appears relatively safe when used in cats (150). IP administration of MSCs was shown to be beneficial also in the treatment of bladder detrusor deterioration in rats (151) and in inflammatory bowel disease in mice (93). Moreover, the IP approach was used to inject Neo-islets, aggregates of ADMSCs and pancreatic islet cells in an FDA guided pilot study in insulin-dependent diabetes mellitus in pet dogs. Neo-islets appear to engraft, redifferentiate, produce insulin, and do not trigger auto- or alloimmune response (152).

## MSC Preconditioning With Proinflammatory Cytokines

Since many patients treated with MSCs suffer from acute or chronic inflammatory diseases, the inflammatory environment is likely to be present *in vivo* when MSCs are being administered. Priming MSCs with IFN-γ before treatment, therefore, imitates the environment in which MSCs will be present in the body. It was proposed that inflammatory conditions enhance the interaction between MSCs and B-cells. Luk et al. (70) showed that MSCs cultured under inflammatory enviroment significantly reduced B-cell proliferation and IgG production by B-cells via induction of indolamine-2,3-dioxygenase activity (IDO), whereas MSCs cultured under non-inflammatory conditions increased the percentage B-regs, but did not influence their proliferation. Tissue origin should also be considered when deciding about priming of MSCs with IFN-γ as, for example, canine MSCs from Wharton jelly are not influenced by IFN-γ (102). In correlation with preconditioning MSCs to improve their therapeutic potential, preconditioning of ECVs has also been shown to be beneficial for their therapeutic effectiveness. Recently it was reported that ECVs from canine MSCs, preconditioned with antiinflammatory cytokines, enhanced macrophage polarization and generation of Tregs in murine colitis (92). However, some studies also reported adverse effects of preconditioning MSC with proinflammatory cytokines. IFN-γ pretreatment enhanced the immunogenicity of MSC with the upregulation of MHC (71) and MHC II expression (153–155). IFN-γ pretreatment also importantly upregulates the expression of genes involved in apoptosis, reflecting negative influence on MSC (156). It was also shown that treatment with both IFN-γ and TNF-α induced apoptosis in mice MSCs. Apoptosis was stimulated by the expression of inducible nitric oxide synthase (iNOS) and the generation of nitric oxide, required for apoptosis (157). To avoid adverse effects of preconditioning with IFN-γ with simultaneous enchancement of their immunosuppressive abilities, pretreatment of MSCs with IL-17A was proposed as an alternative (156). A study by Brandt et al. (158) demonstrated that equine ADMSCs are compromised in an inflammatory environment. High concentrations of proinflammatory cytokines TNF-α and IL-1β and the presence of leukocytes increased ADMSCs proliferation potential and osteogenic differentiation, but negatively affected cells' viability, engraftment, chondrogenic and adipogenic differentiation potential, and expression of the musculoskeletal markers. Conflicting results from various studies about preconditioning MSC with proinflammatory cytokines, therefore, suggest that, although there is a potential beneficial effect of such pretreatments, these should be considered very carefully, and further studies will be needed to clarify potential positive effects of such preconditioning.

## Clinical use of MSC in Veterinary Medicine

To date, stem cells have been used, mostly experimentally, for treatments of a variety of diseases in different animal species. The initial focus of regenerative veterinary medicine was directed to the orthopedic diseases, but the focus is now rapidly expanding to other areas such as orodental and digestive tract diseases, liver, renal, cardiac, respiratory, neuromuscular, dermal, olfactory, and reproductive system diseases. Stem cell treatments were most often used in dogs and horses for various diseases of various organ systems, and in cats for renal, respiratory, and inflammatory diseases.

### Musculoskeletal System Diseases

#### Tendons and Ligaments Diseases

Traumatic and stress injuries of tendons and ligaments naturally heal with the formation of a scar tissue, which is functionally deficient in comparison to the healthy tissue. While the initial injury causes a reduction in structural stiffness, fibrosis obliterates the physiological architecture, and function of the tendon or ligament (159). This results in compromised locomotor function prone to re-injury (160). The optimal treatment should, therefore, aim at the restoring normal structure and function of the tissue. Traditional therapies for tendon injuries in horses are based on cooling (161), bandage, and rehabilitation period with controlled exercise. Pharmacological treatments include the use of systemic and local corticosteroids or other anti-inflammatory drugs (162), but surgical treatment is often required (163, 164). These conservative techniques do not allow for complete tissue healing, reinjury is common, and often animals aren't able to return to the preinjury performance level (162). Ideal treatment should, therefore, aim to regenerate normal tendon matrix. The use of MSC has been introduced as an alternative to the traditional approach because it represents a potential tool for better tissue regeneration (165, 166). Regenerative cell-based therapy aims toward healing with the proper formation of collagen fibers and successful regaining of normal tendon activity with a lesser risk for reoccurrences. It is predicted that MSC isolated from the same tissue that needs treatment would be the most adequate source of MSC for stem cell therapy. The best source of stem cells for tendinopathies would, therefore, be tendon-derived stem cells (167), but the isolation of stem cells from tendon tissue is very challenging, and no standard induction protocol for tendonogenesis exists (168). Stem cells from other sources, mainly from adipose tissue and bone marrow, were therefore used for tendon regeneration. Autologous BMMSC implantation into the horse superficial digital flexor tendon was first reported in 2003 (165). After cells were injected into 11 racehorses with superficial digital flexor tendon lesions, significant clinical recovery was reported (169). Similarly, in a cohort study including 141 racehorses with naturally occurred superficial digital flexor tendon injury, intralesional injection of autologous BMMSC resulted in <28% of reinjuries in all horses with 2 years follow up (170). Results showed a significant reduction in reinjury rate compared to those from a similar study of the same type of injury and follow-up, where horses were treated with intralesional injection of hyaluronan, beta aminopropionitrile fumarate or polysulfated glycosaminoglycans (160). It was demonstrated by Smith et al. (171) that autologous BMMSC treatment of naturally occurring tendinopathies induces the formation of tissue resembling a normal tendon matrix rather than a fibrous tissue that is formed during the natural healing process. In addition to the autologous MSC therapy, promising results were also reported with allogeneic MSC therapy for tendon and ligament disorders such as tendinitis of superficial and deep digital flexor tendons and desmitis of the suspensory and inferior check ligaments (172). However, in surgically induced lesions of the equine superficial digital flexor tendons, autologous BM- or ADMSC therapy rendered no or very small improvement in comparison to other treatments like platelet-rich plasma (PRP) (173, 174).

Similar to horses, dogs were also subjected to experimental MSC treatments. A common injury in dogs is a tear of a cranial crucial ligament in the stifle joint (175). Its rupture is associated with stifle osteoarthritis and is the most common cause of lameness in adult dogs (176). Currently, the recommended therapy is a surgical correction (177).

Positive treatment results from several studies highlighted the value of MSC use in this condition. It was demonstrated that the level of post-operative lameness and pain after single intra-articular injection of allogeneic BMMSC could be a valuable alternative to 1 month course of oral administration of non-steroidal anti-inflammatory drugs (NSAIDs) in dogs requiring tibial plateau leveling osteotomy (TPLO) (178). It was shown that intraarticularly injected autologous BMMSCs engraft to the site of the injured cranial crucial ligament (179) and have an anti-inflammatory effect. Post-operatively intraarticular or IV injection of autologous MSC in dogs with the same condition resulted in a decreased level of CD8+ T-cells, decreased serum and synovia CRP, and decreased synovial IFN-γ levels that persisted over 8 weeks after BMMSC injection (180). In cases of partial tears with no destabilization of the stifle joint, where surgery is not the optimal solution, promising results were collected from the retrospective study, where autologous BMMSC treatment in combination with PRP prevented progression of further degenerative changes in the joint and contralateral ligament rupture in dogs (181).

#### Joint Diseases

Because of the relative hypocellularity and avascularization, cartilage tissue has a limited capacity of self-repair. In horses, it is further affected by the enormous loading forces and mechanical stress that are placed on the articular surfaces during the performance (182). One of the most common reasons for equine athletic career-ending and chronic lameness are joint diseases, with osteoarthritis being the most prevalent (183). Conventional treatment of musculoskeletal injuries, involving the damage to the articular cartilage, ligaments, and menisci is often associated with poor prognosis for the athletic performance of horses (184, 185). The *in vivo* effectiveness of intra-articular MSC treatment of bone, meniscal, and cartilage conditions in horses has been reported. The most studied and described locomotive system disorder in horses is bone spavin, a degenerative joint disease in which conventional treatment is based on the application of anti-inflammatory corticosteroids for decreasing pain and inflammation. Results obtained from the study in which 16 horses with bone spavin were treated intraarticularly with autologous ADMSC suggest the positive and long-lasting effect of MSC therapy. No signs of lameness were observed 180 days after treatment in the treated horses in comparison to the untreated control group. This was confirmed by scintigraphic examination, revealing no signs of inflammation process in tarsal joints of treated horses when compared to the control group where inflammation was still present (186). MSC treatment is also very promising in horses with meniscal damage. Horses treated with intraarticular administration of autologous BMMSC returned to work in a higher percentage than those treated with arthroscopy alone (187). In one study, 80 horses with osteoarthritis were treated with allogeneic ADMSC, and a significant reduction in the lameness was observed during 90 days follow-up period, suggesting the beneficial effect of allogeneic cells (188). Similarly, allogeneic umbilical cord derived MSC for the treatment of osteoarthritis of metacarpophalangeal/metatarsophalangeal joint in horses resulted in a significantly improved lameness over 6 months, but no clinical differences were observed with either single or repeated MSC injection (65). Some studies, however, did report adverse clinical responses after repeated intraarticular injections of allogeneic MSCs in horses with osteoarthritis (60). Even single injections of allogeneic MSCs have been reported to induce mild to moderate local inflammatory signs (64). Several studies in dogs demonstrated that MSC administration into the arthritic joints decrease the patients' discomfort and increase their functional ability. A significant improvement in lameness was confirmed in dogs with stifle osteoarthritis (189) demonstrated by the significantly delayed progression of osteoarthritis in autologous ADMSC treated joints compared to placebo-treated joints. Similar results were reported by Black et al. (190) and Vilar et al. (191) in dogs with hip osteoarthritis. The effect of intraarticular injection of autologous ADMSCs in treating canine osteoarthritis of different joints seems to be long-lasting, as shown in a study with up to 4-year follow-up (192). Significant improvement of MSC therapy for treating osteoarthritis has also been shown with the use of allogeneic ADMSCs. In 74 dogs treated with allogeneic ADMSCs in a prospective, randomized, masked, and placebo-controlled study, no adverse effects were reported, and efficacy in reducing clinical signs was shown in comparison to the placebo group (193). In another extensive study performed on 203 dogs with severe osteoarthritis, causing severe chronic pain, and lameness, results showed excellent improvement in 90% of young dogs and good improvement in 60% of older dogs 10 weeks after the treatment (194). In a dog model of osteoarthritis treated with allogeneic umbilical cord derived MSCs, cartilage repair was demonstrated in the form of cartilage neogenesis, decreased joint fluid content, reduced inflammatory response, and improved healing of the surrounding tissues in comparison to the control untreated group (27). Contrary to study in horses, repeated allogeneic MSC therapy was shown to be safe with only mild and self-limiting inflammatory reactions without adverse effects even 2 years after intraarticular MSC injection (63). MSC therapy of canine osteoarthritis, either autologous or allogeneic, was also tested and proved to be beneficial in combination with PRP or hyaluronic acid (191, 195, 196). In the comparison of ADMSC and PRP treatments in dogs' osteoarthritis, MSC therapy had stronger and more beneficial effects (197).

MSC therapy in treating musculoskeletal disorders has proven remarkably effective, especially in horses with tendon injuries, bone spavin, and meniscal damages, and in dogs with osteoarthritic conditions. Such positive outcomes of MSC therapies are thus decreasing the need for prolonged local or systemic use of anti-inflammatory drugs with their known toxic side effects. However, additional studies are needed to broaden our knowledge on mechanisms of action of MSCs, and especially allogeneic MSCs, as not all studies provided positive results on their safety when used in the therapy. MSC derived ECVs might represent a promising alternative to the allogeneic MSC therapy as they mimic several biological actions of MSCs. ECV therapy has already been tested for treating suspensory ligament injury in a stallion, rendering positive results shown as increased lesion filling, improved angiogenesis, and elasticity of the damaged tendon (111).

### Orodental Diseases

Oral pain and mastication problems can have a major impact on the quality of the animal's life. Many oral diseases can also lead to systemic problems (198). Oral diseases such as dental caries, periodontal disease, permanent tooth loss, oral mucosal lesions, oropharyngeal cancer, and dental trauma are also one of the major public health problems worldwide (199). With the expanding development of regenerative cell therapy, stem cells have attracted interest in the healing of orodental tissues. Studies focus on MSCs immunomodulatory effects to induce regeneration of dental and periodontal tissues, and differentiation potential of MSCs to improve implant strength and bone tissue repair in the alveolar defects. In addition to usual sources of stem cells such as bone marrow and adipose tissue, cells derived from local tissues such as dental pulp stem cells (DPSC) (200–202) or periodontal ligament stem cells (PDLSC) (203, 204) are studied as a therapeutic option in orodental diseases. In experimental dog models, autologous BMMSCs or xenogeneic periodontal ligament MSCs have proven beneficial in periodontal ligament reconstruction, when combined with the growth factors (205), fibrin glue, PRP (206), ephrinB2—a membrane protein regulating bone homeostasis (204) or with a construct of porous biphasic calcium phosphate (203). Allogeneic ADMSCs alone are also capable of inducing periodontal tissue regeneration in the mini pig periodontal defect model (207). For dental pulp regeneration, autologous (200) and allogeneic (201) stem cells from dental pulp or autologous BMMSCs (208) were efficient in dental pulp regeneration in canine models. Although these studies show promise in orodental tissue regeneration, others have reported no beneficial effect of stem cells in dental conditions, such as defects associated with dental implants (209).

Studies conducted on animal models do indeed represent a basis and reference for the use of stem cells and tissue engineering in promoting orodental tissue regeneration. However, extensive research is still needed to prove the efficacy and usefulness of stem cell treatments for orodental problems on actual patients with naturally occurring diseases.

However, very encouraging results are emerging from the MSC treatment of feline chronic gingivostomatitis (FCGS), a painful and debilitating oral condition in cats, characterized by chronic inflammation of gingiva extending to the buccal and caudal oral mucosa. Cats suffering from FCGS are presented with anorexia, oral pain, weight loss, ptyalism, halitosis, and lack of grooming (210). Current treatment options include medications such as corticosteroids (211), cyclosporin (212), and surgical extraction of teeth (213) and have variable response rate and several possible adverse effects (214). Arzi et al. (215) showed that IV treatment with autologous ADMSC resulted in complete clinical and histological resolution or reduction in clinical disease severity in most cats. Immunomodulation of MSC was demonstrated by the normalization of immune cell subsets, serum protein, and cytokine levels. The results of the study also suggested the absence of CD8Io cells as a biomarker to predict the response to MSC therapy (215). Interestingly, allogeneic ADMSCs seem to have lower clinical efficacy in comparison to autologous MSC in treating FCGS (216). The clinical, histologic and systemic response was demonstrated in 70% of cats with FCGS treated with IV administration of allogeneic ADMSC (217).

### Digestive tract Diseases

Inflammatory bowel disease (IBD) is an autoimmune condition with chronic hypersensitivity reaction in the intestinal mucosa of unknown etiology (218). Some dogs are refractory to the traditional lifelong treatments using cyclosporine or steroids (219). Single IV infusion of allogeneic ADMSCs resulted in clinical remission in 9 out of 11 dogs with severe IBD 6 weeks after the treatment together with a significant increase in albumin, cobalamin, and folate levels in the blood (219). IBD is also relatively common in cats with chronic vomiting and diarrhea. In a placebo-controlled blinded study, cats with IBD were treated with allogeneic ADMSC. The owners reported significant improvement or complete resolution of clinical signs in 5 out of 7 cats. In contrast, in cats receiving placebo, no change, or even worsening of the clinical symptoms were reported (220).

Due to their immunomodulatory and anti-inflammatory effects, MSCs seem to be a suitable alternative therapy for dogs and cats with IBD. Results of preliminary studies are promising, but significant follow-up studies and further research is needed to establish MSC treatment as a safe and effective method for treating IBD in animals.

### Liver Diseases

Several studies focused on stem cell treatments of liver disease in dogs. Yan et al. (142) examined the effect of IV administration of autologous ADMSC for artificially induced acute hepatic injury in dogs. ADMSC homed to the liver, levels of liver enzymes in the peripheral blood were reduced, and liver tissue structure was restored after the therapy, indicating a potential for MSC use in liver diseases in pets. MSC were also used in a canine model of liver cirrhosis. IV application of autologous BMMSC significantly decreased the area of the liver fibrosis and improved liver function in the group receiving cells without any adverse side effects (221). Similarly to the IV, IA administration of BMMSC in a canine model of liver fibrosis was shown to be safe, but, interestingly, the effect on reducing levels of the liver enzymes in peripheral blood lasted longer with IA application of MSC (149). Autologous ADMSCs were also used repeatedly IV to treat 10 dogs with degenerative hepatopathy. All animals exhibited significantly improved liver function concerning the decline in hepatic biomarkers after each application in comparison to the control group (222). A clinical case of hepatocutaneous syndrome treated with MSC was also reported. Allogeneic ADMSCs were administered repeatedly either into the liver parenchyma or IV. The dog survival with regressed or limited clinical signs was longer than expected for this disease (223).

Since IV administration of MSCs results in the accumulation of cells in the liver after being cleared from the lungs (122, 142), IV route of the administration seem to be logical for treating liver diseases that are responsive to the MSC therapy in animals. Yet conclusions on the best administration route and also on the MSC efficacy and safety of allogeneic MSCs in treating liver diseases is limited by a low number of studies conducted on actual patients. Therefore, further studies are needed to address these issues.

### Renal Diseases

Chronic kidney disease (CKD) is a common medical condition in geriatric cats and is characterized by chronic tubulointerstitial nephritis, tubular atrophy, and interstitial fibrosis. Currently, renal transplantation is the only therapy that may restore renal function (224).

Stem cell based therapies may, therefore, present less aggressive treatment options. Due to severe side effects and anesthesia associated risks of intrarenal stem cell inoculation, IV application of stem cells is the preferred choice of cell delivery (225, 226). However, IV administration of allogeneic ADMSC in cats with kidney disease was not associated with any side effects, but neither were any short-term improvements in the renal function reported (227, 228). However, in a study conducted by Vidane et al. (226) cats with spontaneous CKD were repeatedly injected IV with allogeneic MSC derived from the feline amniotic membranes, and after the second administration of MSC, significant improvement in the renal function was observed. Specifically, serum creatinine and urine protein concentrations decreased, and urine specific gravity increased. Considerable improvement was also reported in the overall clinical condition of cats, including food intake and social behavior.

Contradictory results from a few studies hinder the conclusion on the suitability of MSC therapy in cats with CKD. Further studies are necessary to determine the possible influence of different factors that might affect the results of MSC therapy in cats with CKD, such as tissue source of MSC, single or repeated administrations of MSCs, and time of application in regard to the stage of the disease. Additionally, too few studies have been conducted with regard to the safety of allogeneic cells in cats, and this will have to be further explored.

### Cardiac Diseases

In human medicine, cardiac stem cell therapies directed toward myocardial repair following the acute or chronic myocardial infarction are being used for several years (229). Primary myocardial infarction is rarely observed in the companion animals (230). However, in large and giant dog breeds, dilated cardiomyopathy is a fairly common disease. Inevitable progression of this disorder leads to the refractory congestive heart failure and death (231). An experimental treatment for this condition was performed in Dobermans with retrograde coronary venous allogeneic ADMSC delivery. Although the treatment was safe, no beneficial effects of stem cell therapy were observed (231). Similarly, treatment of dilated cardiomyopathy with allogeneic cardiosphere-derived cells did not have any beneficial effects after cells were transplanted into the coronary vessels (232). In smaller dog breeds, the most common cardiac disease is the degenerative valvular disease, which is often complicated by ventricular dilation and dysfunction (233). Petchdee and Sompeewong investigated the effect of IV administration of puppy deciduous teeth derived stem cells on the degenerative valvular disease (234). Their results showed an improvement in the left ventricular ejection fraction, but this was a small study, and more studies will be needed to establish any potential positive effects.

### Respiratory Diseases

Respiratory diseases are a common problem also in veterinary medicine. Especially in horses, asthma, comprised of several diseases such as recurrent airway obstruction (RAO) or inflammatory airway disease, is a severe medical condition for which there is no successful treatment available. The disease develops in the presence of moldy hay, dusty straw, and pollens. Horses suffer from frequent coughing, increased respiratory effort at rest, and exercise intolerance. Clinical signs can be controlled by the administration of corticosteroids, bronchodilators, or changing environment. Medications may have adverse side effects, and new therapy options are needed. Barussi et al. (235) studied the effect of the intratracheal application of bone marrow derived mononuclear cells on the course of the respiratory inflammation in horses affected by RAO. Comparison of treatment with single intratracheal administration of autologous cells and oral therapy with dexamethasone showed that bone marrow-derived mononuclear cells improved clinical signs and the inflammatory response in horses suffering from RAO. Levels of IL-10 increased after the cell treatment and were significantly higher than in the control group treated with dexamethasone. The results of this study correlate with positive results of experimental studies with induced respiratory conditions in dogs (236) and cats (237).

### Neuromuscular Diseases and Injuries

One of the most common neuromuscular injuries in both humans and animals are spinal cord injuries (SCI), which often result in a lifelong disabilities (238). In dogs, spinal cord injury could be induced by trauma or herniated vertebral disc. In both pathologies, stem cell treatments were tested with beneficial results. Autologous BMMSC therapy was tested for spontaneous injury of the spinal cord due to spinal trauma in dogs with locally administered cells through hemilaminectomy. Mild to moderate improvements in gait, nociception, and proprioception were observed in some of the animals (238, 239). In another study, allogeneic BMMSCs were combined with the standard medication therapy and this combination induced significantly better improvement in the functional recovery of the patients with traumatic spinal cord injury in comparison to the conventional medication alone (240). Similarly, as in MSC therapy of dogs with traumatic spinal cord injury, positive results of MSC therapy were also observed in acute disc herniation in dogs. Dogs with acute paraplegia had faster locomotor recovery after the epidural application of ADMSCs in comparison to dogs treated with surgical decompression alone (241). However, in dogs with naturally occurring degenerative intervertebral disc disease, transplantation of autologous BMMSC did not affect clinical outcomes, and no regenerative effects were detected in any of the three dog patients (242).

Results of the studies using MSC for the treatment of the traumatic spinal cord injuries and disc herniation in dogs did show some positive effects, but future studies are necessary to find a way to augment currently observed therapeutic effects of the MSC therapies. One possibility is a tissue engineering approach. In one study, constructed canine MSC-derived neural network tissue was transplanted into the spinal cord and resulted in the gradual restoration of paralyzed limb motor function (243). More studies and further developments are therefore needed to establish whether cell therapy and tissue engineering approaches are beneficial for spinal cord injuries, especially in patients with spontaneous injuries where the progress of the disease is often very different from the experimentally induced pathologies.

### Skin Diseases and Wound Healing

Unsuccessful wound healing is often the consequence of a variety of inadequate cellular and molecular mechanisms. It often leads to the persistent, chronic wound, accompanied by the discomfort for the patient. Therefore, for the treatment of chronic wounds with severe inflammation and hyper-plastic response, MSCs might be potentially a viable treating option due to their anti-inflammatory and regenerative potential (244). Several studies in animal models have shown the beneficial effect of MSC treatment in wound healing in goats (245), sheep (246), horses (247), and dogs (248). Significantly improved cutaneous wound healing was also achieved using MSC derived ECV injected locally to treat circular wounds created in dogs (112). In addition to animal models, significant improvement of naturally occurring wounds has been documented in several studies using stem cell therapy. In four horses with naturally occurring infected wounds unresponsive to conventional therapies, peripheral blood stem cells were injected locally and systemically. In all four cases, the positive outcome of the treatment was seen as crusts formation and small scars in the center of the wound, leading to the tissue overgrowth within 4 weeks after treatment (36). Complete healing of the non-healing skin wound was also observed in a filly after repeated local application of heterologous Wharton/s Jelly derived MSC with the use of carboxymethylcellulose gel. The wound healed completely in 5 days (249).

In addition to wound healing, MSCs were also used for the treatment of atopic dermatitis, one of the most common skin diseases in dogs. Contradictory results of two studies using a similar number of IV administrated ADMSC in dogs with atopic dermatitis have been reported. In the first one, no significant improvement of clinical signs or pruritus was observed (250). The second study included 22 dogs with atopic dermatitis, non-responsive to conventional therapy. Pruritus decreased significantly after 1 week and cadesi-04 scores after 1 month after IV administration of allogeneic ADMSC. Remission of clinical signs lasted for at least 6 months, with no adverse side events observed (251).

Several studies in both laboratory animals and clinical veterinary patients suggest that stem cells might be interesting novel therapy to promote chronic wound healing. But as with other diseases, numerous questions remain unanswered and will have to be addressed in future studies before such treatments will enter general clinical practice. Regarding AD in dogs, the data are very limited, and, therefore, it is impossible to predict at the moment whether stem cell treatments might prove to be beneficial in the future.

### Eye Diseases

Stem cell therapy is also investigated in the ophthalmology. Some eye diseases, for example, corneal ulcers are incurable with available methods. Autologous peripheral blood stem cells were used for the treatment of three clinical cases of chronic corneal ulcers and one case of retinal detachment in the horse, all non-responsive to the conventional therapy. Cells were applied either IV or locally into the ophthalmic artery, by subconjunctival injection or in the form of eye drop formulation. All four patients showed significant improvement after treatment, with the restoration of the epithelial surface as well as a decrease in inflammation (37). Subconjunctival administration of autologous BMMSC also led to the improvement of immune mediated keratitis in 3 out of 4 horses, seen as increased corneal clarity, reduced neovascularization of the area, and decreased surface irregularities (252). Immunomodulatory effects of MSC could potentially change the course of equine recurrent uveitis (ERU), as increased expression of IFN-γ by cd4+ T cells from horses with ERU decreased after incubation with ADMSC *in vitro*. Activation of CD4+- T cells was shown to decrease via contact dependent mechanism and PGE2 signaling (253). In cats, MSC therapy was proposed for the treatment of feline eosinophilic keratitis (FEK), as allogeneic ADMSC implanted subconjunctivally showed promising results seen as an effective decrease in the clinical signs of FEK throughout the study (254). In dogs, MSC therapy has been shown as an effective therapeutic alternative for treating *keratoconjunctivitis sicca* (KCS) or dry eye disease. Allogeneic ADMSC implanted locally around the lacrimal gland of both eyes significantly reduced clinical signs with a sustained effect during a study period (255). Similarly, the study by Sgrignoli et al. (256) demonstrated that the expression of KCS markers CD4, IL-6, IL-1, and TNF-α in dogs was decreased significantly 6 months after repeated topical administration of allogeneic ADMSC into the conjunctival sac.

Based on the published studies, MSC therapy holds a great promise in regenerative eye medicine and presents innovative solutions for several eye diseases in animals, such as corneal ulcers, immune mediated and eosinophilic keratitis, recurrent uveitis, and dry keratoconjunctivitis. However, additional blinded prospective studies, especially for recurrent uveitis in horses, are needed to assess the *in vivo* effect of MSC administration more accurately. Continuous scientific research is undoubtedly needed to fully understand the complexity and severity of specific diseases and regenerative effects of stem cells in the eye therapies, which would contribute to bring stem cell therapy closer to translation into clinics.

### Reproductive System Diseases

Many studies are attempting to find treatments for fertility improvement, both for commercial purposes in farm animals and for the translation into human medicine. The goal of restoring fertility with an intraovarian injection of BMMSC was, however, not accomplished, and ovarian function could not be improved or restored in aged mares (257). Similarly, no changes in sperm parameters or fertility rates were observed after intratesticular administration of allogeneic BMMSC in stallions. However, the safety of the procedure at least suggests that such an approach could be theoretically exploited to treat degenerative testicular conditions (258). Interestingly, dog sperms seem to be susceptible to the treatment with ADMSC derived ECV during cryopreservation, as the addition of ECVs reduced the number of damaged sperms decrease of ROS in thawed semen (259). Based on the results from the treatment of other inflammatory conditions, there is a hope that pathologies of reproductive organs will also be susceptible to the MSC treatment. In mares, for example, endometriosis is an incurable degenerative disease of the uterus and is causing substantial economic losses in the equine industry (260). To exploit MSCs immunomodulatory properties in uterine pathologies, endometrial MSCs were investigated and isolated from sows (261), cows (262), ewes (263), goats (264), and mares (265). MSCs delivery to the uterus of mares with endometriosis has already been proposed by Mambelli et al. (266), who demonstrated that MSCs remain in the uterus up to 21 days after intrauterine application. Still, additional studies are needed to assess potential immunomodulatory and anti-inflammatory properties of endometrial MSC and their potential in the treatment of endometriosis (260). In addition to the reproductive system pathologies, MSC therapy is also investigated for the potential treatment of mastitis in farm animals, showing an antiproliferative effect against *Staphylococcus aureus* mediated mastitis in cows (267) and reparative and antifibrotic effect in goat chronic mastitis (268).

## Safety and Regulatory Aspects of Stem Cell Therapies in Veterinary Medicine

The European Medicines Agency's (EMA) Committee for Medicinal Products for Veterinary Use (CVMP) has proposed some basic guidelines for stem cell based medications for veterinary use. Strict microbiological monitoring during the entire manufacturing process from the sourcing of materials to the finished product is essential. Since the use of allogeneic MSC in dogs and horses is increasing, so are the raising questions for manufacturers, authorities, and users. Currently, no specific guidance is available. Safety aspects of extraneous agents concerning veterinary medicinal products are included in the guidelines for the production and control of immunological veterinary medicinal products. In these guidelines is a list of viruses and bacteria for horses and dogs that should not be present in the medicinal products, and this should be adhered to also with the allogeneic MSC. Furthermore, investigations for protozoa may be relevant for dogs and horses depending on the animal region of origin, a prevalent epidemiological situation in their region of origin, and the travel history of the animal donors. Furthermore, as a general guideline, it is recommended that cell donors are always clinically healthy. If cells from newborn animals or placental tissues are used, it is advisable to test mothers for the presence of any infectious agents. To demonstrate the absence of disease-causing agents. A combination of donor screening using anamnesis and clinical information, donor testing for the presence of specific disease agents and product (cells ready for therapy) testing should be applied. All material with biological origin needed for collection, selection, culture, and modification of cells should also be clearly specified and evaluated for the absence of any potentially harmful agents. Furthermore, aseptic manufacturing is necessary for reducing the presence of extraneous agents (269). Within the EU, there is currently no central legislation about stem cell therapies in veterinary medicine, so currently, each EU member state regulates the field independently. However, this is expected to change in the near future with EMA issuing guidelines and legislative rules for regenerative veterinary medicine.

In 2015 the USA Food and Drug Administration (FDA) published recommendations for the use of cell-based products in animals. According to this, cell-based products, including animal stem cell based products (ASCP) that are intended for use in the diagnosis, mitigation, treatment, or prevention of diseases, are regulated as new animal medicines and require a premarket review to be legally marketed. The requirements for approval include the demonstration of safety, effectiveness, and manufacturing quality. Evaluation of tumorigenicity, immunogenicity, donor selection criteria, the transmission of infectious agents, long term safety, cell survival, biodistribution, and ectopic tissue formation are required (48). In the future, additional regulatory guidelines can be expected. It is unclear whether these new regulations will significantly affect the advancement of stem cell trials and the development of novel therapies (230), but any new regulations should be prepared and approved by experts from various fields, from cell biology to clinical veterinary medicine. Currently, no animal cell based treatments are FDA-approved. Considering a great promise that veterinary regenerative medicine holds for the future, FDA started the Veterinary Innovation Program or VIP to help manufacturers/providers of stem cell therapies with obtaining high-quality data from well-conducted, well-controlled, and well-designed scientific studies (270).

## Summary

Veterinary regenerative medicine is an active area of research. Significant advances in developing safe and effective stem cell therapies have been made in recent years. Notable outcomes of MSC therapies have been reported, especially for orthopedic conditions in dogs and horses, but important advancements in MSC therapy have also been made in treating other conditions such as FCGS, IBD, and wound healing. Positive outcomes of many studies suggest a great promise for the future of stem cell therapies for various animal diseases, but numerous issues need to be addressed. One of them is the optimal source for MSC isolation. Adipose and bone marrow derived MSC were used in the majority of the studies, but mostly because they are easily obtainable and easy to work with. Therefore, other stem cells from different tissues might prove in the future to be more suitable for the treatments of certain diseases.

Furthermore, the effect of age and potentially sex on the medicinal properties of MSC will have to be established in future studies. Time with regard to the disease progression, dosage of cells, and mode of MSC application also vary widely between studies. There are no standard protocols established that would suggest the optimal treatment protocols for specific diseases. IV application of MSCs has been often used for treating various animal diseases, despite some suggestions about short viability and rapid clearance of cells. However, some studies suggest that healing immunomodulatory processes in the body are induced by apoptosis-mediated immunomodulation through the immune cells, and this could prolong the action of MSCs. Lung entrapment of MSC after IV application is also an important issue in the field of systemic stem cell therapies. Although other routes of administration have been considered to avoid lung entrapment, the main alternative to IV administration of MSC might be systemic administration of ECV. Early studies suggest that ECVs could be a promising, cell-free stem cell therapy that would prevent lung entrapment and avoid possible pulmonary embolism caused by IV application of MSC, but further studies are needed about both efficacy and safety of ECVs. Another unresolved question is the use of autologous or allogeneic cells. Autologous cells are certainly safer, but their use is more complicated and expensive for the animal owners. Allogeneic cells from healthy donors are, therefore, a possible alternative, but there are still unresolved questions about their immunogenicity and potential to trigger an immune response in the recipient of cells.

Despite considerable advancements in veterinary regenerative medicine in recent years, this field is still in its infancy and much more work is needed to resolve many questions before proven, standardized therapies could be offered to the clinical patients. We live in exciting times as new regenerative therapies are on the rise. One can be hopeful that the continuous research in this area will lead us to the point when the stem cell treatments for many currently untreatable diseases will not be a mere possibility but a realistic and accessible choice for the patients in both veterinary and human medicine.

## Author Contributions

MVo and NA drafted the manuscript. MVe and GM edited the draft. All authors contributed to the final manuscript.

## Conflict of Interest

GM is partial owner of Animacel ltd. The authors declare that the research was conducted in the absence of any commercial or financial relationships that could be construed as a potential conflict of interest.
